# The Association between Mutational Signatures and Clinical Outcomes among Patients with Early-Onset Breast Cancer

**DOI:** 10.3390/genes15050592

**Published:** 2024-05-07

**Authors:** Robert B. Basmadjian, Dylan E. O’Sullivan, May Lynn Quan, Sasha Lupichuk, Yuan Xu, Winson Y. Cheung, Darren R. Brenner

**Affiliations:** 1 Department of Community Health Sciences, Foothills Medical Centre, University of Calgary, Calgary, AB T2N 4Z6, Canada; 2 Department of Oncology, Tom Baker Cancer Centre, University of Calgary, Calgary, AB T2N 4N2, Canada; 3 Department of Cancer Epidemiology and Prevention Research, Cancer Research and Analytics, Cancer Care Alberta, Alberta Health Services, Calgary, AB T2S 3C3, Canada; 4 Department of Surgery, Foothills Medical Centre, University of Calgary, Calgary, AB T2N 2T9, Canada

**Keywords:** early-onset, young age, breast cancer, mutational signatures, genomic sequencing, cancer bioinformatics, biomarkers, biostatistics, patient outcomes, personalized medicine

## Abstract

Early-onset breast cancer (EoBC), defined by a diagnosis <40 years of age, is associated with poor prognosis. This study investigated the mutational landscape of non-metastatic EoBC and the prognostic relevance of mutational signatures using 100 tumour samples from Alberta, Canada. The MutationalPatterns package in R/Bioconductor was used to extract de novo single-base substitution (SBS) and insertion–deletion (indel) mutational signatures and to fit COSMIC SBS and indel signatures. We assessed associations between these signatures and clinical characteristics of disease, in addition to recurrence-free (RFS) and overall survival (OS). Five SBS and two indel signatures were extracted. The SBS13-like signature had higher relative contributions in the HER2-enriched subtype. Patients with higher than median contribution tended to have better RFS after adjustment for other prognostic factors (HR = 0.29; 95% CI: 0.08–1.06). An unsupervised clustering algorithm based on absolute contribution revealed three clusters of fitted COSMIC SBS signatures, but cluster membership was not associated with clinical variables or survival outcomes. The results of this exploratory study reveal various SBS and indel signatures may be associated with clinical features of disease and prognosis. Future studies with larger samples are required to better understand the mechanistic underpinnings of disease progression and treatment response in EoBC.

## 1. Introduction

Breast cancer remains the most diagnosed malignancy and most common cause of cancer death among women globally, with an estimated 2.3 million diagnoses and 666,000 deaths in 2022 [1]. Approximately 4.5% of cases in Canada are early-onset breast cancer (EoBC), defined by a diagnosis before 40 years of age, compared to an estimated 10% of cases worldwide [1,2]. In Canada, the incidence rate of EoBC has increased annually by 0.66% from 2000 to 2015 compared to 0.21% in the overall population during the same time period [3]. Further, survival outcomes have not improved in EoBC to the same extent as in the overall breast cancer population over time [4].

EoBC presents clinical challenges in part due to its rarity but also because of few established risk factors for prevention. Organized routine mammography screening in Canada is indicated for women aged 50–74 years, with the harms of screening outweighing the benefits in women < 40 years [5]. Therefore, EoBC is often detected symptomatically and at later stages [6,7,8,9]. EoBC is also likely to present with more aggressive disease biology, including the human epidermal growth factor receptor-2 (HER2)-enriched and triple-negative (TNBC) subtypes, stressing the importance of the clinical management of EoBC [10,11]. It is well accepted that the risk of recurrence and mortality is higher in EoBC compared to the overall breast cancer population; however, drivers remain poorly understood. Large epidemiological studies established age < 40 years as an independent risk factor of poor prognosis in breast cancer, even after adjustment of pathological features and treatments received [4,10,12,13,14,15,16,17]. This has driven clinical debate as to whether inferior outcomes in EoBC are due to an overrepresentation of aggressive disease features or a unique disease biology [18].

The strongest established risk factors in EoBC include inherited genetic mutations. However, less than 10% of breast cancer incidence among young women is attributable to heritable mutations in the *BRCA1* or *BRCA2* genes [19,20]. Further, Copson et al. found no evidence that germline mutations were related to mortality or tumour aggressiveness among breast cancer patients aged < 40 [19]. This suggests an important role for somatic mutations caused by lifestyle or environmental exposures in combination with intrinsic processes in tumour progression and survival in young women. Somatic mutations are found in all cancer genomes. A small proportion are drivers that confer clonal advantage, are causally implicated in oncogenesis, and have been positively selected during the evolution of the cancer [21,22,23,24]. Somatic driver mutations in over 30 cancer genes have been implicated in breast cancer development, including *AKT1*, *BRCA1*, *CDH1*, *GATA3*, *PIK3C*, *PTEN*, *RB1*, and *TP53* [10,21,22]. Comparatively fewer studies have assessed driver mutations of recurrence and metastasis in breast cancer, and no such studies have been performed in early-onset populations.

The remaining somatic mutations are “passengers”, which do not contribute to cancer development. However, passenger mutations bear the imprints of the DNA damage and repair processes operative during the development of the cancer, unmodified by selection [25]. Advancements in next-generation sequencing have permitted sequencing of whole cancer genomes and identified thousands of single nucleotide variants (SNVs) in breast cancer genomes [26,27]. There are six unique types of SNVs: C>A, C>G, C>T, T>A, T>C, and T>G. Each of the substitutions is examined by incorporating information on the bases immediately 5′ and 3′ to each mutated base generating 96 possible mutation types (6 types of substitution*4 types of 5′ base*4 types of 3′ base). The array of mutation types is represented in a mutational spectrum, then decomposed into recurring patterns, referred to as mutational signatures. Sixty validated single-base substitution (SBS) mutational signatures are listed in the Catalogue Of Somatic Mutations In Cancer (COSMIC) version 3.3, in addition to 18 insertion–deletion (indel) signatures [28]. Mutational signatures can be used to decipher how patterns of somatic mutations collectively give rise to mutational processes of disease as well as give insight into the potential etiology of the processes underlying these signatures.

Mealey et al. performed one of the most comprehensive analyses of the mutational landscape of breast cancer ≤40 years [23]. They found that COSMIC signatures SBS1, 3, and 5 were the most common in the overall cohort and that SBS2 and SBS3 were more likely to be observed in HER2-enriched and triple-negative tumours, respectively. Compared to patients >60 years, early-onset patients were significantly more likely to have C>A mutations (17% vs. 16%) and less likely to have C>T mutations (32% vs. 38%). Finally, patients ≤40 years were more likely to have mutations in *GATA3* compared to those >40 years and >60 years (22% vs. 12.9% vs. 10.8%) [23]. Studies like this provide insight into multiple genomic features related tumour development in women < 40 years. To date, there have been no applications of mutational signatures to assess outcomes in EoBC and no studies have investigated indel signatures among these patients. Similar to Mealey et al., genomic data can be leveraged to understand how various somatic mutations collectively drive tumour progression and survival in young women. These analyses may discover novel markers to inform targeted therapies or may improve the performance of existing prediction tools to better inform individualized prognosis. In this study, we examine whole exome sequences from 100 EoBC patients in Alberta, Canada to describe their somatic mutation landscape, including mutational load, SBS, and indels. We also extracted de novo SBS and indel signatures and fit mutational profiles to validate COSMIC SBS and indel signatures. Finally, we examined whether extracted and fitted COSMIC signatures were associated with clinicopathological tumour characteristics and survival outcomes.

## 2. Materials and Methods

### 2.1. Study Sample and Data Collection

Somatic mutation and clinical data were obtained from 100 women between the ages of 18–39 years diagnosed with invasive non-metastatic breast cancer in Alberta, Canada, from 2001 to 2014. Mutational data were derived from tumour tissue and normal blood samples stored at the Alberta Cancer Registry Biobank. Tumour tissue was extracted at time of surgery or biopsy and stored as formalin-fixed paraffin embed blocks. Blood samples were also collected at time of surgery or biopsy and centrifuged for buffy coat extraction. Tumour and normal blood samples were sent to Genome Québec for DNA extraction and whole-exome sequencing. Extraction was performed with QIAsymphony DSP DNA Kits (QIAGEN, Hilden, Germany) and sequencing was performed with NovaSeq 6000 S4 PE100 (Illumina, San Diego, USA) and SureSelect Human All Exon exome probes (Agilent Technologies, Santa Clara, USA). Following sequencing, variant calling was performed using the Mutect2 workflow [29] from the Canadian Centre for Computational Genomics (C3G) and obtained in the form of variant call files (VCF). The corresponding reference genome was GRCh37/hg19. Clinical data were obtained through linkage with the Alberta Cancer Registry and included detailed information on baseline demographics, cancer diagnosis (stage and morphology), dates of referral to oncology, clinic visits at any of the cancer centers, surgical procedures, dates and types of therapy received for cancer (chemotherapy, radiation, and hormonal therapy), tumour size, grade, lymph node status, ER/PR status and HER2/neu status, and dates of last follow-up or death. Administrative end of follow-up was 25 February 2018.

### 2.2. Extraction of Mutational Signatures

Mutational signatures were investigated using the MutationalPatterns package in R (v4.3)/Bioconductor (v3.17) [30]. This package includes a comprehensive set of functions for extracting mutational signatures de novo and determining the contribution of previously identified mutational signatures on a single sample level. The package works with SNVs, indels, double-base substitutions (DBS) and larger multi-base substitutions (MBS). The VCF files for each participant were passed through “read_vcfs_as_granges” and “get_mut_type” commands to obtain counts of the six SNV types (C>A, C>G, C>T, T>A, T>C, T>G) and indel types.

De novo SBS and indel mutational signature extraction was achieved with non-negative matrix factorization (NMF) using the “extract_signatures” command. The NFM algorithm is detailed by Gaujoux and Seoighe [31]. In brief, the algorithm factorizes some matrix *X*, which has rows *n* and columns *m*, into two smaller nonnegative matrices *W* and *H*, where the product of *W* and *H* approximates *X*. *W* is defined by *n* × *r* and *H* is defined by *r* × *m*, where *r* is the factorization rank, which is the number of extracted de novo signatures. We sampled ranks from 2 to 10. The optimal factorization rank was based on the smallest rank for which the cophenetic correlation coefficient started decreasing. For example, in the case of SBS mutations, the rows of matrix *X* were the 96 mutational contexts derived from combinations of 6 mutational types (i.e., C>A, C>G, C>T, T>A, T>C, and T>G) and their 5′- and 3′-adjacent bases, and the columns were the 100 EoBC samples. The optimal rank can be interpreted as the minimal set of mutational signatures that optimally explains the proportion of each mutation type and estimates the contribution of each signature to each sample [32]. The “fit_to_signature” command determined which COSMIC SBS and indel signatures were present in our samples. This function finds the optimal linear combination of mutation signatures that most closely reconstructs the mutation matrix by solving the nonnegative least-squares constraints problem. 

### 2.3. Statistical Analysis

All demographic, clinical, pathological, and mutation data were described using means and standard deviations (SD) for continuous variables and frequency tables with proportions for categorical variables. The means of mutational load (the sum of SNV and indel mutations) and relative contribution of de novo SBS and indel signatures were compared across categories of patient characteristics using Welch’s two-sample T-test. These variables included age at diagnosis (<30, 30–34, ≥35 years), BMI category (underweight or normal [<25 kg/m^2^], overweight [25–29.99 kg/m^2^], obese [≥30 kg/m^2^]), patient-reported family history of breast cancer (no, yes), molecular subtype (luminal, HER2-enriched, TNBC), ER status (negative, positive), PR status (negative, positive), HER2 status (negative, positive), lymph node status (negative, positive), positive lymph node count (0, 1–3, ≥4), tumour size (≤2 cm, >2 cm), T stage (T1, T2, T3, T4), tumour grade (low, high), and presence of lymphovascular invasion (negative, positive). 

As there are 60 and 18 validated COSMIC SBS and indel signatures, respectively, we employed hierarchal clustering algorithms to determine specific combinations of mutational signature contributions. This clustering analysis was only performed on COSMIC signatures present in >25% of samples. Absolute contribution values for each signature were standardized prior to clustering. Euclidian distance was then calculated to form a distance matrix and passed through a hierarchal clustering algorithm based on Ward’s minimum variance method. The average silhouette method determined the optimal number of clusters. The unadjusted associations between cluster membership and demographic and clinical variables were assessed with Fisher’s exact test and multivariable logistic regression assessed mutually adjusted associations. 

Recurrence-free survival (RFS) and overall survival (OS) were the primary outcomes to evaluate the prognostic relevance of de novo signatures and COSMIC signature clusters. RFS was defined as time from primary surgery to local–regional or distant relapse, contralateral breast cancer, the appearance of a second (non-breast) primary tumor, or death from breast cancer. OS was defined as time from primary surgery to death from any cause. De novo signatures were converted into binary variables based on absolute contribution below the median (low expression), or equal to or greater than the median (high expression). The Kaplan–Meier method was used to estimate curves for RFS and OS, as well as median time-to-event and 95% confidence intervals (95% CI). Association measures were estimated with multivariable Cox proportional hazard models in the form of hazard ratios (HR) with 95% CI. Statistical significance was defined by *p*-value <0.05. All analyses were performed in RStudio (v2023.06.0+421). 

## 3. Results

### 3.1. Cohort Characteristics

Demographic and clinical characteristics of the 100 EoBC cases included in this study are presented in Table 1. The mean age of diagnosis was 33.8 years (SD = 4.54) and 21% were diagnosed before age 30 years, 24% were 30–34 years, and 55% were 35–39 years. Almost half (48%) of the cohort had a positive family history of breast cancer. Less than half of the cohort was classified as overweight (32%) and obese (15%). Luminal disease was the most common (57%) subtype in this cohort followed by HER2-enriched (28%) and TNBC (15%). The majority of cases were lymph node-negative (54%), high grade (53%), larger than 2 cm (53%), and involved lympho-vascular invasion (65%).

### 3.2. Mutational Load

The median mutational load (SNVs + indels) identified in EoBC tumours was 596.5 (IQR = 478.25–688.25). Mutational load was primarily comprised of SNVs, with a median of 567 (IQR = 469.25–657.75). The median number of indels was 26 (IQR = 20.75–34.00). The distributions of mutational load, number of SNVs, and number of indels were positively skewed so the data were log-transformed to examine differences across demographic and clinical variables. In general, mean of the log-transformed mutational load and number of SNVs tended to be higher in the overweight BMI category versus normal/underweight, TNBC subtype versus luminal, lymph node-negative tumours versus lymph node-positive, and tumours ≤2 cm versus >2 cm, although statistical significance was not achieved (0.05 ≥ *p*-value < 0.20) (Table 2). Those without vascular invasion had significantly higher mean log-transformed mutational load (*p* = 0.007) and number of SNVs (*p* = 0.009) versus those with vascular invasion (Table 2). Regarding log-transformed indels, the mean was significantly higher in the TNBC subtype versus luminal (*p* = 0.029), lymph node-negative tumours (*p* = 0.035), and in those without vascular invasion (*p* = 0.001) (Table 2).

### 3.3. Extracted De Novo SBS and Indel Signatures

**Figure 1:**
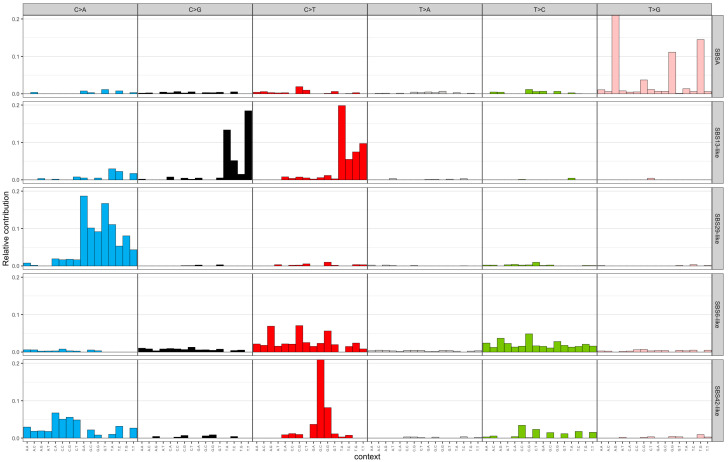
Extracted single-base substitution signatures from 100 early-onset breast cancer patients in Alberta, Canada using non-negative matrix factorization. The x-axis represents trinucleotide context (5′ and 3′ nucleotides) for the six SNV types (C>A, C>G, C>T, T>A, T>C, T>G) and the y-axis represents relative contribution. SBS = single-base substitution; SNV = single-nucleotide variant.

**Figure 2:**
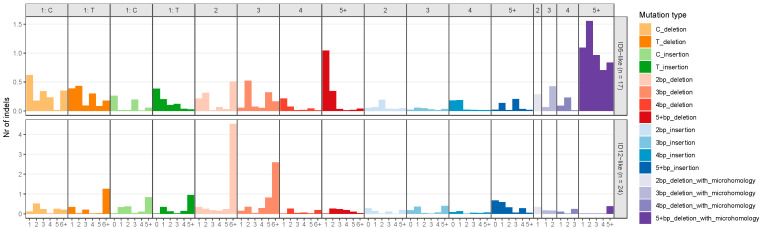
Extracted insertion–deletion signatures from 100 early-onset breast cancer patients in Alberta, Canada using non-negative matrix factorization. The x-axis represents the homopolymer length for single-base pair deletions and insertions, the number of repeat units for >1 base pair deletions and insertions at repeats, and microhomology length for microhomology deletions. The y-axis is the number of insertions–deletions.

The NMF algorithm decomposed the mutational spectra of all breast tumours into five SBS and two indel signatures. These signatures were named after existing COSMIC signatures if they had a cosine similarity of more than 0.85. The SBS signatures were named as follows: SBSA, SBS13-like, SBS29-like, SBS6-like, and SBS42-like. Figure 1 illustrates the distribution of SNV types for the extracted SBS signatures. SBSA did not have a cosine similarity of 0.85 with any existing COSMIC signature and was characterized by a high contribution of T>G mutations. The SBS13-like signature had high relative contributions from C>T and C>G mutations. The SBS29-like signature comprised of C>A mutations. Low peaks of C>T and T>C mutations defined the SBS6-like signature. Finally, the SBS42-like signature had high relative contribution of C>T mutations, followed by C>A and T>C mutations. The two de novo indel signatures were named ID6-like and ID12-like as they had a cosine similarity of more than 0.85 with existing COSMIC signatures (Figure 2). The ID6-like signature had a high frequency of microhomology deletions of ≥5 base pairs and the ID12-like signature had high frequency of >1 base pair deletions at repeat sites. 

Table 3 compares mean relative contribution of the de novo SBS and indel signatures across categories of clinical variables. For SBS13-like, mean relative contribution was significantly higher in those aged 30–34 (*p* = 0.026) and 35–39 (*p* = 0.015) relative to <30 years, higher in the HER2-enriched subtype relative to luminal (*p* = 0.034), and T3 tumours relative to T1 (*p* = 0.011). The mean relative contribution of the SBS29-like signature was significantly lower in the TNBC subtype than luminal (*p* < 0.001). Relative to the normal/underweight BMI category, the overweight BMI category had significantly lower mean relative contribution of the SBS6-like (*p* = 0.003) and SBS42-like signatures (*p* = 0.045). As there were only two extracted indel signatures, the relative contributions of ID6-like and ID12-like were complimentary. The mean relative contribution of the ID6-like signature was significantly higher in the obese BMI group versus normal/underweight and significantly lower in the HER2-enriched subtype versus luminal. Relative contribution plots for the de novo SBS and indel signatures are presented in Figures S1 and S2. 

Table 4 presents crude and mutually adjusted HR estimates for the associations between each de novo signature and RFS, as well as OS. In general, there was no evidence to conclude whether the hazard of recurrence differed between high-expression and low-expression groups for most signatures. However, the unadjusted HR for the SBS13-like signature demonstrated a significant reduction in recurrence hazard for those with high signature expression versus low expression (HR = 0.36; 95% CI: 0.13–0.98). The mutually adjusted estimate showed a 71% reduction (HR = 0.29; 95% CI: 0.08–1.06), although statistical significance was not achieved. Similar reductions in the hazard of death were estimated for the SBS13-like signature but with less precision. 

### 3.4. COSMIC SBS and Indel Signatures and Clustering Analysis

**Figure 3:**
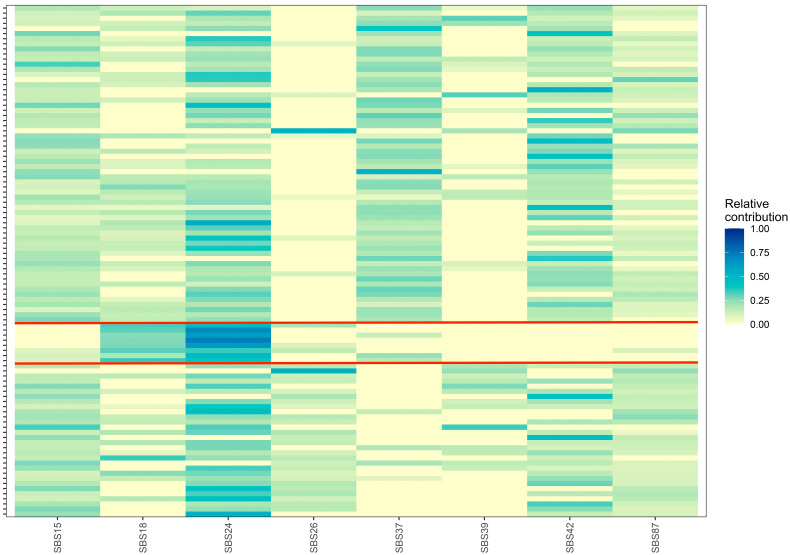
Relative contribution heatmap of unsupervised hierarchal clustering analysis of eight COSMIC single-base substitution signatures into three distinct clusters, which are separated by red lines. The x-axis represents the COSMIC single-base substitution signatures and the y-axis represents samples. SBS = single-base substitution.

The mean relative contribution and prevalence of all COSMIC SBS and indel signatures in our 100 EoBC cases are presented in Tables S1 and S2, and relative contribution plots are presented in Figures S3 and S4, respectively. Six SBS signatures were present in over 50% of the cohort: SBS15 (91%), SBS24 (89%), SBS87 (77%), SBS42 (76%), SBS13 (67%), and SBS18 (60%). Twenty-six SBS signatures were not present. All COSMIC indel signatures were present. ID12 was present in 98% of the cohort and had a mean relative contribution of 50% (SD = 20.4%), the highest of all COSMIC signatures. In total, eight COSMC SBS signatures had a prevalence of >25% and were included in the hierarchal clustering algorithm: SBS15, SBS18, SBS24, SBS26, SBS37, SBS39, SBS42, SBS87. Three clusters were identified (Figure 3). Cluster 1 (*n* = 62) included contributions from all signatures except SBS26 and SBS39. Cluster 2 (*n* = 8) included substantial contributions from SBS18 and SBS24. Cluster 3 (*n* = 30) included contributions from all signatures except SBS37. We did not observe statistically significant univariable associations between cluster membership and clinical variables (Table 5). Evidence was insufficient to conclude whether the unadjusted and mutually adjusted hazards of recurrence and death of Cluster 2 and 3 differed from Cluster 1 (Table 6). We attempted to perform hierarchal clustering with the COSMIC indel signatures; however, due to high relative contribution of ID12 in most samples, only one cluster was identified.

## 4. Discussion

In this study, we characterized the somatic mutation landscape of 100 EoBC tumours from Alberta, Canada and assessed their relationship with clinicopathological tumour features and survival outcomes. Our findings indicated higher numbers of SNVs and indels among patients without vascular invasion, in addition to a higher number of indels with lymph node-negative and TNBC tumours. We extracted five de novo SBS signatures, four of which resembled validated COSMIC SBS signatures, and two de novo indel signatures resembling ID6 and ID12. The mean relative contribution of these de novo signatures mainly differed between BMI categories and molecular subtypes. RFS tended to be better among individuals with high SBS13-like signature expression relative to low, and worse in those with high SBS29-like signature expression relative to low. The hierarchal clustering algorithm of validated COSMIC SBS signatures revealed three distinct clusters. However, evidence was insufficient to conclude whether cluster membership was associated with clinical variables and with survival outcomes. 

This is the first study to examine the prognostic relevance of somatic mutational signatures and describe differences in signature distribution across clinicopathological tumour characteristics among patients with EoBC. We expanded upon previous work from Mealey et al., who investigated differences in mutational profiles between breast cancer patients < 40 years and ≥40 years with The Cancer Genome Atlas (TCGA) Breast Invasive Carcinoma project (TCGA-BRCA) data [23]. They also extracted five de novo SBS signatures in their <40 years subgroup, three of which had similar SNV mutation profiles to the signatures we extracted. Specifically, SBSA, SBS6-like, and SBS13-like signatures in our study resembled signatures S2, S3, and S1 in their study, respectively. SBSA had high relative contributions of T>G in the ATG, TTG, and GTT contexts. This was visually most alike COSMIC SBS55, previously observed in Alexandrov et al., a non-validated signature arising from a possible sequencing artifact. The SBS6-like signature was characterized by low peaks of C>T and T>C mutations. The peaks of C>T mutations in the ACG, CCG, and GCG contexts were similar to COSMIC SBS1 and SBS6, and the contribution of T>C mutations likely reflects a combination of signatures present at low levels. The SBS29-like and SBS42-like signatures were unique to this study and are generally not found in breast cancers [28]. COSMIC SBS29 is linked to chewing tobacco use and SBS42 is linked to haloalkane exposure. The role of smokeless tobacco in breast cancer is not well established. A hospital-based case-control study in Assam, India, found the odds of being diagnosed with breast cancer were 2.35 times higher in betel quid chewers vs. non-chewers [33]. Interestingly, SBS29 was also found among early-onset testicular cancer tumours, although there is no established link between chewing tobacco and testicular cancer. It is possible that SBS29 represents the process involved in early-onset cancers, but greater research is needed in other sites to confirm this speculation. 

The SBS13-like signature resembled a combination of COSMIS SBS2 and SBS13, which often occur together in the same sample. These signatures are attributed to the activity of the AID/APOBEC family of cytidine deaminases, which substantially contribute to the mutation burden in many human cancers, especially in bladder and breast cancers [32]. We observed higher relative contributions of the SBS13-like signature in the HER2-enriched subtype and HER2-positive tumours, similar to Mealey et al. [23]. Further, our findings show RFS and OS tended to be better in patients with high SBS13-like expression, even after adjustment for the subtype. Among breast cancer subtypes, HER2+ breast tumours are reported to have the highest median levels of APOBEC signature enrichment [34]. APOBEC-related mutagenesis is thought to play an important role in tumour immunogenicity, namely in neoantigen presentation and recruitment of T-cells to the tumour microenvironment, implying its potential for cancer immunotherapy [35]. However, this likely depends on the molecular subtype. In a TCGA cohort, DiMarco et al. observed high correlation between APOBEC enrichment and immune signatures reflective of an antitumor adaptive immune response in the TNBC subtype, including Th1 cells, CD8^+^ T cells, cytotoxic cells, interferon signaling pathway, major histocompatibility complex class II antigen presentation pathway [36]. Conversely, the APOBEC enrichment score was not correlated with immune cell signatures in HER2-enriched breast cancers. Instead, APOBEC enrichment was associated with a higher frequency of subclonal mutations and may suggest the evolution of immune-suppressive mechanisms that limit antitumor adaptive immune responses [36]. These findings suggest a subgroup of TNBC patients who may benefit from immunotherapy and equally a subgroup of HER2+ patients who may not benefit from immunotherapy beyond anti-HER2 therapy. Unfortunately, our prognostic findings of the SBS13-like signature could not be stratified by subtype due to limited sample size and we could not ascertain if these effects were mediated by treatment received. Nonetheless, there may be a role of ABOPEC-related mutational signatures, like SBS2 and SBS13, as a biomarker for immunotherapy response in breast cancer, regardless of age. APOBEC signatures are associated with a greater likelihood of response to immune checkpoint inhibition in non-small cell lung cancer, head and neck cancer, and bladder cancer [32,36,37,38]. 

We also extracted de novo indel signatures that resembled COSMIC ID6 and ID12. Currently, the proposed etiology of the ID12 signature is unknown. The ID6 signature arises from defective homologous recombination-based DNA damage repair, often due to inactivating *BRCA1* or *BRCA2* mutations, leading to non-homologous DNA end-joining activity [32]. Given that these mutations are associated with younger age and TNBC, it was not unexpected that this signature was extracted in our EoBC cohort, and that relative contribution was highest in the TNBC subtype. Further, we found that the number of indel mutations was higher in TNBCs. Although the ID6-like signature did not bear prognostic significance in our study, there is an important role for homologous recombination deficiency (HRD) in TNBC. Poly(ADP-ribose) polymerases (PARP) inhibitors have been successfully implemented in the treatment of metastatic breast cancer with germline mutations in *BRCA1/2* [39,40]. The recent OlympiA trial also established the efficacy of PARP inhibitors for *BRCA1/2* mutation carriers in the early-stage setting, where the median age of the trial population was 43 years, and 82% of participants had TNBC [41]. The application of these treatments is being explored in patients who display a “BRCAness” phenotype. BRCAness refers to malignancies that have not arisen from germline *BRCA1* or *BRCA2* mutations but share the phenotypic and molecular features of HRD [42]. These malignancies share the same therapeutic vulnerabilities with *BRCA*-associated tumors including sensitivity to platinum chemotherapy [43,44,45]. However, there is no standardized biomarker of “BRCAness” currently available. Further characterization of this phenotype may aid in predicting response to PARP inhibitors in expanded patient populations. 

Our analysis of fitting mutational profiles to COSMIC SBS signatures revealed results not in line with previous literature. This is the first study to examine COSMIC v3.2 signatures in the EoBC setting; therefore, these analyses were exploratory in nature. We found high prevalence of newly added signatures, including SBS37, SBS39, SBS42, and SBS87. The most common COSMIC signatures previously observed in breast tumours are SBS1, SBS2, SBS3, SBS5, SBS13, and SBS18. Mealey et al. found that SBS1, SBS3, and SBS5 were the most prevalent COSMIC signatures and had the highest mean contributions in patients < 40 years. Conversely, we observed each of these signatures in five or fewer patients. We observed SBS13 in 15% of samples and SBS18 in 60% of samples. Given that our extracted de novo SBS signatures matched similar profiles to those from Mealey et al. and Nik-Zainal et al. [23,46], these discrepancies may be explained by suboptimal fitting of known COSMIC signatures rather than biological differences between study samples. The MutationalPattern package uses COSMIC v3.2 whereas Mealey et al. was based on COSMIC v2.0 [23]. It is possible that doubling the number of signatures led to overfitting and misattribution in our sample. That is, if samples contained various combinations of mutational signatures the fitting algorithm may erroneously attribute mutations to one signature. This may explain why we did not observe any associations between the COSMIC SBS cluster group and clinical variables. Therefore, we cannot confidently conclude that the high prevalence of recently added COSMIC SBS signatures is biologically or clinically relevant in EoBC.

This study included several strengths. To our knowledge, it is the first to investigate the prognostic relevance of SBS and indel signatures EoBC. We examined multiple characterizations of somatic mutations including mutation load, SNVs, indels, and mutational signatures. Further, provide information on their associations with important molecular and physical tumour characteristics, as well as with RFS and OS. We also extracted an APOBEC-like SBS signature in EoBC, consistent with previous findings, and elucidated extracted indel signatures. There are several limitations to note. First, our study included small sample size, limiting the statistical power and generalizability of our results. Second, this study used WES data so we cannot draw conclusions related to mutations in the genome outside the exome. We also did not investigate germline mutations or signaling pathways, and so did not produce new evidence linking mutational signatures to germline mutations or cellular signaling. Third, the exploratory nature of the study meant the use of data-driven techniques. For example, we converted extracted SBS and indel signatures to binary variables based on a median cut-off for the survival analyses. We also used an unsupervised clustering algorithm for COSMIC SBS signatures. Although these methods have been used in previous research, we cannot confirm their clinical or biological relevance. Fourth, due to the limited sample size, we lacked sufficient power to examine the prognostic relevance of signatures within subgroups and we did not have data on patient race and ethnicity. Mutational profiles can vary between racial and ethnic groups and may explain disparities in therapeutic response and cancer outcomes. 

## 5. Conclusions

Drivers of poor outcomes in EoBC are an active area of ongoing research. In addition to identifying cancer etiologies and the causes of driver mutations, analysis of mutational signatures can also lead to direct therapeutic and prognostic insights. An increasing number of bioinformatics studies show how mutational signatures may predict response to immunotherapy, as well as bear imprints of DNA damage from chemotherapy and radiation treatment that may accelerate disease progression [47]. The results of this exploratory study reveal various SBS and indel signatures may be associated with clinical variables of disease and prognosis. Future studies with larger samples are required to better understand the mechanistic underpinnings of disease progression and treatment response in EoBC.

## Figures and Tables

**Table 1:** Patient characteristics of the study sample, which included 100 patients diagnosed with invasive breast cancer 18–39 years of age in Alberta from 2001 to 2014.

Characteristic	Overall (*n* = 100)
Age at diagnosis (years)	
Mean (SD)	33.80 (4.54)
Median [IQR]	35 [31, 38]
<30	21 (21.0%)
30–<35	24 (24.0%)
≥35	55 (55.0%)
Body mass index (kg/m^2^)	
Mean (SD)	25.58 (5.48)
Median [IQR]	24.51 [21.66, 28.26]
Normal/under (<25)	53 (53.0%)
Overweight (25–29.99)	32 (32.0%)
Obese (≥30)	15 (15.0%)
Family history of breast cancer	
No	43 (43.0%)
Yes	48 (48.0%)
Missing	9 (9.0%)
Invasive type	
Ductal	89 (89.0%)
Lobular	3 (3.0%)
Mixed	1 (1.0%)
Other	6 (6.0%)
Missing	1 (1.0%)
Presence of vascular invasion	
No	33 (33.0%)
Yes	65 (65.0%)
Missing	2 (2.0%)
Overall grade	
Low	19 (19.0%)
High	53 (53.0%)
Missing	28 (28.0%)
Lymph node status	
Negative	54 (54.0%)
Positive	45 (45.0%)
Missing	1 (1.0%)
Number of positive lymph nodes	
Mean (SD)	1.62 (2.99)
Median [IQR]	0 [0, 2]
Zero	54 (54.0%)
1 to 4	31 (31.0%)
5 or more	14 (11.0%)
Missing	1 (1.0%)
Total Invasive Tumour Size	
Mean (SD)	2.57 (2.64)
Median [IQR]	2 [1, 3]
≤2 cm	36 (36.0%)
>2 cm	53 (53.0%)
Missing	11 (11.0%)
T stage	
T1	49 (49.0%)
T2	45 (45.0%)
T3	3 (3.0%)
T4	3 (3.0%)
HER2 status	
Negative	72 (72.0%)
Positive	28 (28.0%)
ER status	
Negative	21 (21.0%)
Positive	79 (79.0%)
PR status	
Negative	40 (40.0%)
Positive	60 (60.0%)
Molecular subtype	
Luminal	57 (57.0%)
HER2-enriched	28 (28.0%)
TNBC	15 (15.0%)
Chemotherapy	
Did not receive	5 (5%)
Received	95 (95%)
Anti-HER2 therapy	
Did not receive	72 (72%)
Received	28 (28%)
Hormone therapy	
Did not receive	44 (44%)
Received	56 (56%)
Radiation therapy	
Did not receive	31 (31%)
Received	69 (69%)

Abbreviations: cm = centimetre; ER = estrogen receptor; HER2 = human epidermal growth factor receptor 2; IQR = interquartile range; kg = kilograms; m = metres; PR = progesterone receptor; SD = standard deviation; T stage = tumour stage; TNBC = triple-negative breast cancer.

**Table 2:** Comparing the mean of the log of mutational load, number of single nucleotide variants, and number of insertion–deletion mutations across categories of patient characteristics.

	Log Transformed Mutational Load (Mean (SD))		
Characteristics	SNV + Indels	SNVs Only	Indels Only
Age category (years)			
<30	6.38 (0.29)	6.34 (0.30)	3.20 (0.34)
30–34	6.34 (0.36)	6.29 (0.36)	3.34 (0.42)
≥35	6.37 (0.39)	6.33 (0.40)	3.23 (0.47)
*p*-value <30 vs. 30–34	0.694	0.632	0.306
*p*-value <30 vs. ≥35	0.897	0.875	0.815
Body mass index (kg/m^2^)			
Normal/under (<25)	6.33 (0.43)	6.28 (0.43)	3.26 (0.47)
Overweight (25–29.99)	6.43 (0.28)	6.39 (0.28)	3.17 (0.38)
Obese (≥30)	6.38 (0.26)	6.33 (0.28)	3.41 (0.38)
*p*-value normal vs. overweight	0.199	0.165	0.368
*p*-value normal vs. obese	0.601	0.636	0.232
Family history of breast cancer			
No	6.38 (0.40)	6.34 (0.40)	3.19 (0.42)
Yes	6.36 (0.34)	6.31 (0.34)	3.32 (0.42)
*p*-value	0.724	0.653	0.134
Molecular subtype			
Luminal	6.34 (0.42)	6.30 (0.42)	3.21 (0.46)
HER2-enriched	6.34 (0.23)	6.30 (0.24)	3.22 (0.26)
TNBC	6.51 (0.35)	6.46 (0.35)	3.48 (0.52)
*p*-value Luminal vs. HER2	0.990	0.998	0.912
*p*-value Luminal vs. TNBC	0.120	0.135	0.029
ER status			
Negative	6.44 (0.34)	6.39 (0.34)	3.41 (0.47)
Positive	6.35 (0.37)	6.30 (0.37)	3.21 (0.42)
*p*-value	0.281	0.309	0.089
HER2 status			
Negative	6.38 (0.41)	6.33 (0.41)	3.26 (0.49)
Positive	6.34 (0.23)	6.30 (0.24)	3.22 (0.26)
*p*-value	0.683	0.687	0.635
Lymph node status			
Negative	6.43 (0.43)	6.38 (0.43)	3.34 (0.49)
Positive	6.30 (0.26)	6.25 (0.27)	3.15 (0.33)
*p*-value	0.067	0.075	0.035
Number of positive lymph nodes			
Zero	6.43 (0.43)	6.38 (0.43)	3.34 (0.49)
1 to 3	6.32 (0.28)	6.27 (0.28)	3.16 (0.36)
4 or more	6.24 (0.19)	6.20 (0.20)	3.14 (0.28)
*p*-value zero vs. 1–3	0.141	0.155	0.056
*p*-value zero vs. 4 or more	0.120	0.124	0.173
Tumour size category			
2 cm or less	6.44 (0.39)	6.39 (0.39)	3.35 (0.46)
more than 2 cm	6.27 (0.34)	6.23 (0.34)	3.13 (0.39)
*p*-value	0.120	0.125	0.182
T stage			
T1	6.38 (0.44)	6.34 (0.45)	3.27 (0.52)
T2	6.36 (0.28)	6.31 (0.28)	3.27 (0.33)
T3	6.34 (0.10)	6.30 (0.09)	2.93 (0.39)
T4	6.22 (0.27)	6.17 (0.27)	3.06 (0.23)
*p*-value T1 vs. T2	0.756	0.757	0.996
*p*-value T1 vs. T3	0.831	0.879	0.190
*p*-value T1 vs. T4	0.450	0.465	0.418
Tumour grade			
High	6.36 (0.33)	6.31 (0.33)	3.28 (0.37)
Low	6.34 (0.54)	6.30 (0.54)	3.17 (0.54)
*p*-value	0.827	0.86	0.349
Presence of vascular invasion			
No	6.50 (0.43)	6.45 (0.43)	3.43 (0.46)
Yes	6.29 (0.31)	6.25 (0.31)	3.14 (0.37)
*p*-value	0.007	0.009	0.001

Abbreviations: cm = centimetre; ER = estrogen receptor; HER2 = human epidermal growth factor receptor 2; kg = kilograms; m = metres; SD = standard deviation; T stage = tumour stage; TNBC = triple-negative breast cancer.

**Table 3:** Comparing the mean relative contribution of extracted de novo single-base substitution and insertion–deletion mutational signatures across categories of patient characteristics. Relative contribution is a proportion between 0 and 1.

	Extracted Signatures (Mean (SD))						
Characteristics	SBSA	SBS13-like	SBS29-like	SBS6-like	SBS42-like	ID6-like	ID12-like
Age category (years)							
<30	0.21 (0.13)	0.11 (0.06)	0.24 (0.17)	0.20 (0.07)	0.24 (0.08)	0.44 (0.20)	0.56 (0.20)
30–34	0.20 (0.12)	0.18 (0.13)	0.17 (0.07)	0.21 (0.08)	0.23 (0.07)	0.48 (0.20)	0.52 (0.20)
≥35	0.19 (0.11)	0.18 (0.16)	0.20 (0.12)	0.20 (0.11)	0.22 (0.08)	0.44 (0.18)	0.56 (0.18)
*p*-value <30 vs. 30–34	0.851	0.026	0.087	0.634	0.922	0.539	0.539
*p*-value <30 vs. ≥35	0.571	0.015	0.358	0.868	0.591	0.948	0.948
Body mass index (kg/m^2^)							
Normal/under (<25)	0.19 (0.13)	0.14 (0.10)	0.20 (0.12)	0.23 (0.12)	0.24 (0.08)	0.44 (0.18)	0.56 (0.18)
Overweight (25–29.99)	0.22 (0.12)	0.20 (0.19)	0.21 (0.15)	0.17 (0.07)	0.21 (0.08)	0.41 (0.19)	0.59 (0.19)
Obese (≥30)	0.19 (0.05)	0.1 (0.09)	0.17 (0.06)	0.22 (0.06)	0.23 (0.05)	0.58 (0.20)	0.42 (0.20)
*p*-value normal vs. overweight	0.303	0.114	0.820	0.003	0.049	0.431	0.431
*p*-value normal vs. obese	0.906	0.162	0.258	0.772	0.700	0.027	0.027
Family history of breast cancer							
No	0.21 (0.13)	0.16 (0.15)	0.20 (0.11)	0.20 (0.11)	0.23 (0.07)	0.44 (0.18)	0.56 (0.18)
Yes	0.19 (0.11)	0.16 (0.13)	0.21 (0.15)	0.21 (0.09)	0.22 (0.08)	0.46 (0.19)	0.55 (0.19)
*p*-value	0.553	0.965	0.662	0.829	0.850	0.582	0.582
Molecular subtype							
Luminal	0.20 (0.12)	0.14 (0.11)	0.22 (0.13)	0.21 (0.11)	0.23 (0.08)	0.47 (0.19)	0.53 (0.19)
HER2-enriched	0.17 (0.11)	0.22 (0.19)	0.19 (0.13)	0.19 (0.08)	0.23 (0.09)	0.35 (0.13)	0.65 (0.13)
TNBC	0.24 (0.12)	0.17 (0.08)	0.15 (0.04)	0.23 (0.08)	0.22 (0.04)	0.58 (0.23)	0.42 (0.23)
*p*-value Luminal vs. HER2	0.278	0.034	0.318	0.319	0.867	0.100	0.001
*p*-value Luminal vs. TNBC	0.314	0.290	<0.001	0.466	0.526	0.001	0.100
ER status							
Negative	0.23 (0.13)	0.17 (0.08)	0.17 (0.13)	0.22 (0.09)	0.22 (0.06)	0.50 (0.23)	0.50 (0.23)
Positive	0.19 (0.11)	0.17 (0.15)	0.21 (0.12)	0.20 (0.10)	0.23 (0.08)	0.44 (0.18)	0.56 (0.18)
*p*-value	0.255	0.945	0.154	0.432	0.511	0.231	0.231
HER2 status							
Negative	0.21 (0.12)	0.14 (0.11)	0.20 (0.12)	0.21 (0.10)	0.23 (0.07)	0.49 (0.20)	0.51 (0.20)
Positive	0.17 (0.11)	0.22 (0.19)	0.19 (0.13)	0.19 (0.08)	0.23 (0.09)	0.35 (0.13)	0.65 (0.13)
*p*-value	0.159	0.043	0.588	0.206	0.948	<0.001	<0.001
Lymph node status							
Negative	0.20 (0.12)	0.18 (0.15)	0.19 (0.13)	0.21 (0.12)	0.22 (0.08)	0.47 (0.20)	0.53 (0.20)
Positive	0.20 (0.12)	0.15 (0.11)	0.21 (0.11)	0.20 (0.07)	0.24 (0.07)	0.43 (0.17)	0.57 (0.17)
*p*-value	0.986	0.204	0.357	0.548	0.145	0.274	0.274
Number of positive lymph nodes							
Zero	0.20 (0.12)	0.18 (0.15)	0.19 (0.14)	0.21 (0.12)	0.22 (0.08)	0.47 (0.20)	0.53 (0.20)
1 to 3	0.22 (0.11)	0.14 (0.12)	0.22 (0.13)	0.19 (0.07)	0.23 (0.07)	0.45 (0.16)	0.55 (0.16)
4 or more	0.14 (0.11)	0.16 (0.10)	0.21 (0.07)	0.22 (0.07)	0.27 (0.07)	0.37 (0.19)	0.63 (0.19)
*p*-value zero vs. 1–3	0.465	0.186	0.373	0.390	0.460	0.587	0.587
*p*-value zero vs. 4 or more	0.141	0.579	0.570	0.827	0.044	0.129	0.129
Tumour size category							
2 cm or less	0.20 (0.11)	0.16 (0.12)	0.21 (0.14)	0.20 (0.12)	0.22 (0.08)	0.44 (0.20)	0.56 (0.20)
more than 2 cm	0.19 (0.12)	0.16 (0.15)	0.20 (0.13)	0.21 (0.09)	0.24 (0.08)	0.47 (0.19)	0.55 (0.19)
*p*-value	0.610	0.973	0.741	0.781	0.323	0.405	0.405
T stage							
T1	0.21 (0.13)	0.16 (0.12)	0.19 (0.13)	0.21 (0.12)	0.22 (0.08)	0.44 (0.20)	0.56 (0.20)
T2	0.18 (0.11)	0.17 (0.15)	0.22 (0.15)	0.20 (0.07)	0.24 (0.08)	0.47 (0.18)	0.53 (0.18)
T3	0.21 (0.03)	0.11 (0.01)	0.23 (0.04)	0.18 (0.08)	0.26 (0.03)	0.44 (0.29)	0.56 (0.29)
T4	0.31 (0.15)	0.20 (0.22)	0.10 (0.08)	0.17 (0.04)	0.22 (0.10)	0.35 (0.15)	0.65 (0.15)
*p*-value T1 vs. T2	0.152	0.912	0.281	0.602	0.392	0.507	0.507
*p*-value T1 vs. T3	0.960	0.011	0.180	0.623	0.190	0.999	0.999
*p*-value T1 vs. T4	0.370	0.810	0.177	0.326	0.936	0.413	0.413
Tumour grade							
High	0.19 (0.12)	0.17 (0.15)	0.20 (0.13)	0.21 (0.09)	0.23 (0.07)	0.45 (0.20)	0.55 (0.20)
Low	0.17 (0.10)	0.12 (0.08)	0.26 (0.16)	0.21 (0.14)	0.25 (0.09)	0.42 (0.18)	0.58 (0.18)
*p*-value	0.389	0.065	0.157	0.948	0.438	0.473	0.473
Presence of vascular invasion							
No	0.19 (0.11)	0.15 (0.14)	0.22 (0.16)	0.22 (0.12)	0.21 (0.09)	0.47 (0.22)	0.53 (0.22)
Yes	0.20 (0.12)	0.17 (0.14)	0.19 (0.11)	0.20 (0.09)	0.24 (0.07)	0.43 (0.17)	0.57 (0.17)
*p*-value	0.906	0.431	0.346	0.359	0.158	0.320	0.320

Abbreviations: cm = centimetre; ER = estrogen receptor; HER2 = human epidermal growth factor receptor 2; kg = kilograms; m = metres; SD = standard deviation; T stage = tumour stage; TNBC = triple-negative breast cancer.

**Table 4:** Estimated hazard ratios for the relationships between extracted de novo single-base substitution mutational signatures, insertion–deletion mutational signatures, and both recurrence-free and overall survival.

	Recurrence-Free Survival				Overall Survival			
Signature *	Crude HR (95% CI)	*p*-Value	Adjusted HR (95% CI) ª	*p*-Value	Crude HR (95% CI)	*p*-Value	Adjusted HR (95% CI) º	*p*-Value
SBSA		0.687		0.755		0.864		0.329
Low	1.00 (ref)		1.00 (ref)		1.00 (ref)		1.00 (ref)	
High	0.83 (0.34–2.03)		1.22 (0.34–4.40)		0.90 (0.26–3.11)		2.33 (0.43–12.71)	
SBS13-like		0.045		0.063		0.148		0.161
Low	1.00 (ref)		1.00 (ref)		1.00 (ref)		1.00 (ref)	
High	0.36 (0.13–0.98)		0.29 (0.08–1.06)		0.37 (0.09–1.43)		0.29 (0.05–1.63)	
SBS29-like		0.344		0.095		0.935		0.889
Low	1.00 (ref)		1.00 (ref)		1.00 (ref)		1.00 (ref)	
High	1.54 (0.63–3.79)		3.08 (0.92–11.5)		1.05 (0.30–3.64)		0.89 (0.19–4.30)	
SBS6-like		0.525		0.395		0.176		0.596
Low	1.00 (ref)		1.00 (ref)		1.00 (ref)		1.00 (ref)	
High	1.33 (0.55–3.23)		1.72 (0.49–6.02)		2.55 (0.66–9.89)		1.62 (0.27–9.61)	
SBS42-like		0.507		0.856		0.654		0.353
Low	1.00 (ref)		1.00 (ref)		1.00 (ref)		1.00 (ref)	
High	0.73 (0.30–1.83)		0.87 (0.18–4.13)		0.75 (0.21–2.63)		0.48 (0.10–2.28)	
ID6-like		0.185		0.699		0.977		0.777
Low	1.00 (ref)		1.00 (ref)		1.00 (ref)		1.00 (ref)	
High	0.54 (0.21–1.35)		0.79 (0.24–2.59)		0.98 (0.28–3.40)		0.79 (0.17–3.82)	
ID12-like		0.424		0.593		0.873		0.957
Low	1.00 (ref)		1.00 (ref)		1.00 (ref)		1.00 (ref)	
High	1.46 (0.58–3.67)		1.47 (0.36–5.97)		0.90 (0.26–3.13)		1.05 (0.18–6.16)	

* High expression means absolute contribution was equal to or above the median. Low expression means absolute contribution was below the median. ª Adjusted for age category, BMI category, molecular subtype, tumour size category, lymph node count, and grade. º Adjusted for age category, BMI category, lymph node count, ER status, and grade. Abbreviations: CI = confidence interval; ID = insertion–deletion; HR = hazard ratio; SBS = single-base substitution.

**Table 5:** Comparing the frequency of patients in cluster groups resulting from the unsupervised hierarchal clustering algorithm of COSMIC single-base substitution signatures across categories of patient characteristics.

	COSMIC SBS Cluster			
Characteristics	Cluster 1 (*n* = 62)	Cluster 2 (*n* = 8)	Cluster 3 (*n* = 30)	*p*-Value
Age category (years)				0.431
<30	12 (19.4%)	4 (50.0%)	5 (16.7%)	
30–34	35 (56.5%)	3 (37.5%)	17 (56.7%)	
≥35	15 (24.2%)	1 (12.5%)	8 (26.7%)	
Body mass index (kg/m^2^)				0.363
Normal/under (<25)	32 (51.6%)	3 (37.5%)	18 (60.0%)	
Overweight (25–29.99)	10 (16.1%)	0 (0%)	5 (16.7%)	
Obese (≥30)	20 (32.3%)	5 (62.5%)	7 (23.3%)	
Family history of breast cancer				0.855
No	27 (43.5%)	3 (37.5%)	13 (43.3%)	
Yes	28 (45.2%)	5 (62.5%)	15 (50.0%)	
Molecular subtype				0.137
Luminal	38 (61.3%)	7 (87.5%)	12 (40.0%)	
HER2	16 (25.8%)	1 (12.5%)	11 (36.7%)	
TNBC	8 (12.9%)	0 (0%)	7 (23.3%)	
ER status				0.378
Negative	11 (17.7%)	1 (12.5%)	9 (30.0%)	
Positive	51 (82.3%)	7 (87.5%)	21 (70.0%)	
HER2 status				0.384
Negative	46 (74.2%)	7 (87.5%)	19 (63.3%)	
Positive	16 (25.8%)	1 (12.5%)	11 (36.7%)	
Lymph node status				0.573
Negative	33 (53.2%)	3 (37.5%)	18 (60.0%)	
Positive	28 (45.2%)	5 (62.5%)	12 (40.0%)	
Number of positive lymph nodes				0.818
Zero	33 (53.2%)	3 (37.5%)	18 (60.0%)	
1 to 3	20 (32.3%)	4 (50.0%)	7 (23.3%)	
4 or more	8 (12.9%)	1 (12.5%)	5 (16.7%)	
Tumour size category				0.309
2 cm or less	35 (56.5%)	7 (87.5%)	14 (46.7%)	
more than 2 cm	21 (33.9%)	1 (12.5%)	11 (36.7%)	
T stage				0.233
T1	34 (54.8%)	4 (50.0%)	11 (36.7%)	
T2	22 (35.5%)	4 (50.0%)	19 (63.3%)	
T3	3 (4.8%)	0 (0%)	0 (0%)	
T4	3 (4.8%)	0 (0%)	0 (0%)	
Tumour grade				0.398
High	31 (50.0%)	3 (37.5%)	19 (63.3%)	
Low	11 (17.7%)	3 (37.5%)	5 (16.7%)	
Presence of vascular invasion				0.146
No	17 (27.4%)	5 (62.5%)	11 (36.7%)	
Yes	43 (69.4%)	3 (37.5%)	19 (63.3%)	

Abbreviations: cm = centimetre; ER = estrogen receptor; HER2 = human epidermal growth factor receptor 2; kg = kilograms; m = metres; SBS = single-base substitution; T stage = tumour stage; TNBC = triple-negative breast cancer.

**Table 6:** Estimated hazard ratios for the relationships between cluster groups resulting from the unsupervised hierarchal clustering algorithm of COSMIC single-base substitution signatures, and both recurrence-free and overall survival.

	Recurrence-Free Survival				Overall Survival			
SBS Cluster	Crude HR (95% CI)	*p*-Value	Adjusted HR (95% CI) ª	*p*-Value	Crude HR (95% CI)	*p*-Value	Adjusted HR (95% CI) º	*p*-Value
1	1.00 (ref)		1.00 (ref)		1.00 (ref)		1.00 (ref)	
2	1.55 (0.35–6.90)	0.567	1.16 (0.25–5.41)	0.852	2.86 (0.59–13.84)	0.193	1.77 (0.34–9.24)	0.5
3	0.92 (0.32–2.60)	0.868	0.86 (0.30–2.44)	0.772	0.37 (0.05–3.03)	0.356	0.32 (0.09–2.65)	0.292

ª Adjusted for age category, BMI category, molecular subtype, tumour size category, lymph node count, and grade. º Adjusted for age category, BMI category, lymph node count, ER status, and grade. Abbreviations: CI = confidence interval; HR = hazard ratio; SBS = single-base substitution.

## Data Availability

The data that support the findings of this study were used under license and ethics approval for this study and are stored on a secure (firewall protected) Alberta Health Services server that complies with institutional requirements for data security. Therefore, the data are not publicly available. However, data may be made available upon reasonable request.

## Supplementary Materials

The following supporting information can be downloaded at: https://www.mdpi.com/article/10.3390/genes15050592/s1, Table S1: The mean relative contribution and proportion of fitted COSMIC single-base substitution mutational signatures in the overall study sample, Table S2: The mean relative contribution and proportion of fitted COSMIC insertion–deletion mutational signatures in the overall study sample, Figure S1: Plot of the relative contribution of extracted de novo single-base substitution mutational signatures in each sample, Figure S2: Plot of the relative contribution of extracted de novo insertion–deletion mutational signatures in each sample, Figure S3: Plot of the relative contribution of fitted COSMIC single-base substitution mutational signatures in each sample, Figure S4: Plot of the relative contribution of fitted COSMIC insertion–deletion mutational signatures in each sample.
