# Supplementary material for: The Association between Mutational Signatures and Clinical Outcomes among Patients with Early-Onset Breast Cancer
Source: Genes (Basel). 2024 May 7;15(5):592. doi: 10.3390/genes15050592 (PMC11121604; doi:10.3390/genes15050592)
Supplement: Supplementary file 1 [file genes-15-00592-s001.zip › genes-2983152-supplementary.pdf]

SUPPLEMENTAL FILE

LIST OF TABLES OF FIGURES

Table S1. The mean relative contribution and proportion of fitted COSMIC single-base substitution mutational signatures in the overall study sample.

Table S2. The mean relative contribution and proportion of fitted COSMIC insertion-deletion mutational signatures in the overall study sample.

Figure S1. Plot of the relative contribution of extracted *de novo* single-base substitution mutational signatures in each sample.

Figure S2. Plot of the relative contribution of extracted *de novo* insertion-deletion mutational signatures in each sample.

Figure S3. Plot of the relative contribution of fitted COSMIC single-base substitution mutational signatures in each sample.

Figure S4. Plot of the relative contribution of fitted COSMIC insertion-deletion mutational signatures in each sample.

Table S1. The mean relative contribution and counts of fitted COSMIC single-base substitution mutational signatures in the overall study sample.

Signature	Absent	Present[a]	Relative contribution*	
			Mean	Standard deviation
SBS1	97	3	0.003314109	0.020484192
SBS2	84	16	0.012650149	0.035955869
SBS3	97	3	0.005267358	0.030410316
SBS4	98	2	0.001974985	0.014585579
SBS5	95	5	0.013121055	0.064628252
SBS6	94	6	0.006827635	0.029423721
SBS7a	100	0	0	0
SBS7b	99	1	0.000455854	0.004558537
SBS7c	100	0	0	0
SBS7d	100	0	0	0
SBS8	100	0	0	0
SBS9	98	2	0.002178937	0.017504073
SBS10a	100	0	0	0
SBS10b	95	5	0.002700745	0.013329969
SBS10c	100	0	0	0
SBS10d	100	0	0	0
SBS11	100	0	0	0
SBS12	99	1	0.000497408	0.004974084
SBS13	85	15	0.01580875	0.048890527
SBS14	100	0	0	0
SBS15	9	91	0.12805659	0.063021744
SBS16	100	0	0	0
SBS17a	84	16	0.006707185	0.015786962
SBS17b	100	0	0	0
SBS18	40	60	0.08146849	0.082422608
SBS19	99	1	0.001316283	0.013162825
SBS20	94	6	0.00417017	0.016857751
SBS21	100	0	0	0
SBS22	100	0	0	0
SBS23	98	2	0.001405123	0.009999708
SBS24	11	89	0.202178023	0.117142943
SBS25	99	1	0.001027989	0.010279894
SBS26	65	35	0.040848974	0.061630382
SBS28	100	0	0	0
SBS29	75	25	0.038918736	0.078531388
SBS30	99	1	0.000560875	0.005608748

SBS31	100	0	0	0
SBS32	100	0	0	0
SBS33	96	4	0.001534403	0.007621185
SBS34	100	0	0	0
SBS35	100	0	0	0
SBS36	99	1	0.000752531	0.007525312
SBS37	33	67	0.114497099	0.091815995
SBS38	91	9	0.004948182	0.016370802
SBS39	72	28	0.033865854	0.06230237
SBS40	99	1	0.00217212	0.021721204
SBS41	100	0	0	0
SBS42	24	76	0.14541753	0.114457707
SBS44	100	0	0	0
SBS84	99	1	0.000854588	0.00854588
SBS85	100	0	0	0
SBS86	100	0	0	0
SBS87	23	77	0.077587249	0.05241288
SBS88	100	0	0	0
SBS89	79	21	0.033454472	0.07655681
SBS90	100	0	0	0
SBS91	81	19	0.011004319	0.026860473
SBS92	100	0	0	0
SBS93	100	0	0	0
SBS94	98	2	0.00245623	0.017372932

*Relative contribution is expressed as a proportion between 0 and 1.
[a]Present means signature has absolute contribution greater than 0.

Table S2. The mean relative contribution and counts of fitted COSMIC insertion-deletion mutational signatures in the overall study sample.

Signature	Absent	Present[a]	Relative contribution*	
			Mean	Standard deviation
ID1	67	33	0.022991837	0.03993133
ID2	37	63	0.045069193	0.05069426
ID3	85	15	0.020575552	0.05608897
ID4	50	50	0.06268373	0.07775451
ID5	93	7	0.010339185	0.03976018
ID6	53	47	0.090744371	0.13145529
ID7	84	16	0.014201936	0.03567362
ID8	59	41	0.066143241	0.09989677
ID9	96	4	0.00321063	0.01610203
ID10	60	40	0.058290291	0.08238785
ID11	81	19	0.019559304	0.04493681
ID12	2	98	0.500130522	0.20391816
ID13	85	15	0.011329714	0.03269677
ID14	84	16	0.012909992	0.03171142
ID15	96	4	0.004832203	0.02582451
ID16	71	29	0.027648472	0.0494149
ID17	77	23	0.026126032	0.05682638
ID18	99	1	0.003213796	0.03213796

*Relative contribution is expressed as a proportion between 0 and 1.

[a]Present means signature has absolute contribution greater than 0.

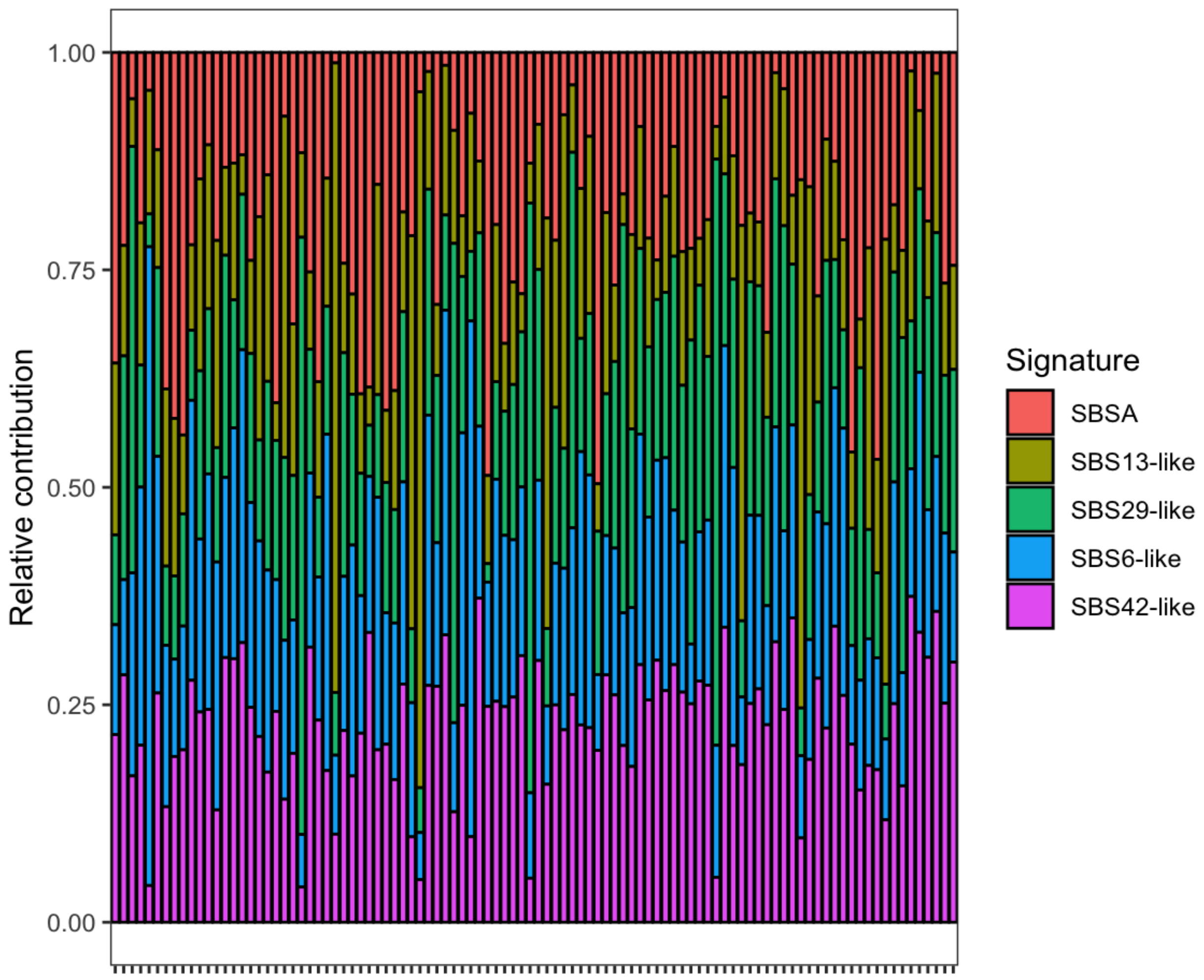

Figure S1. Plot of the relative contribution of extracted *de novo* single-base substitution mutational signatures in each sample.

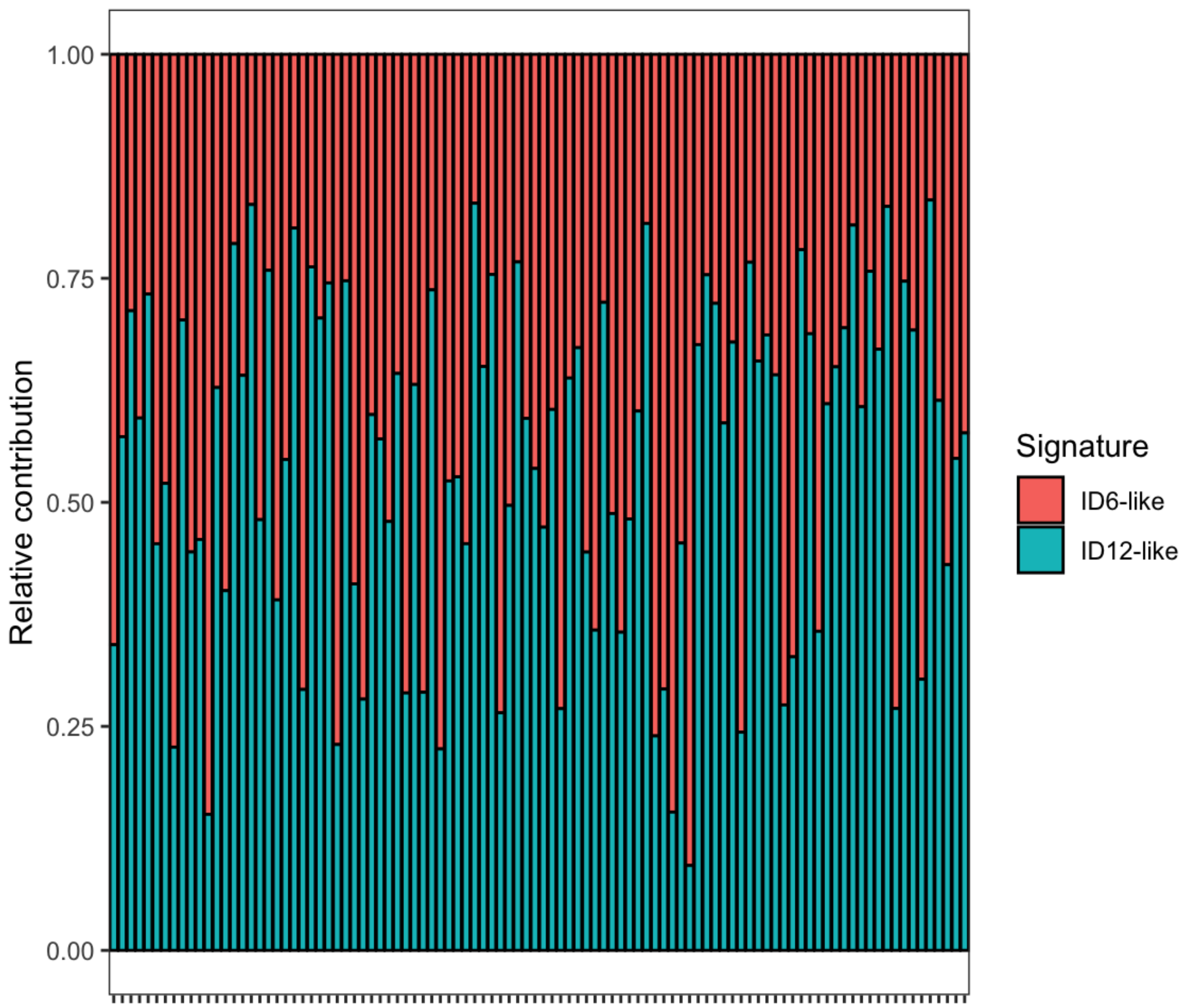

Figure S2. Plot of the relative contribution of extracted *de novo* insertion-deletion mutational signatures in each sample.

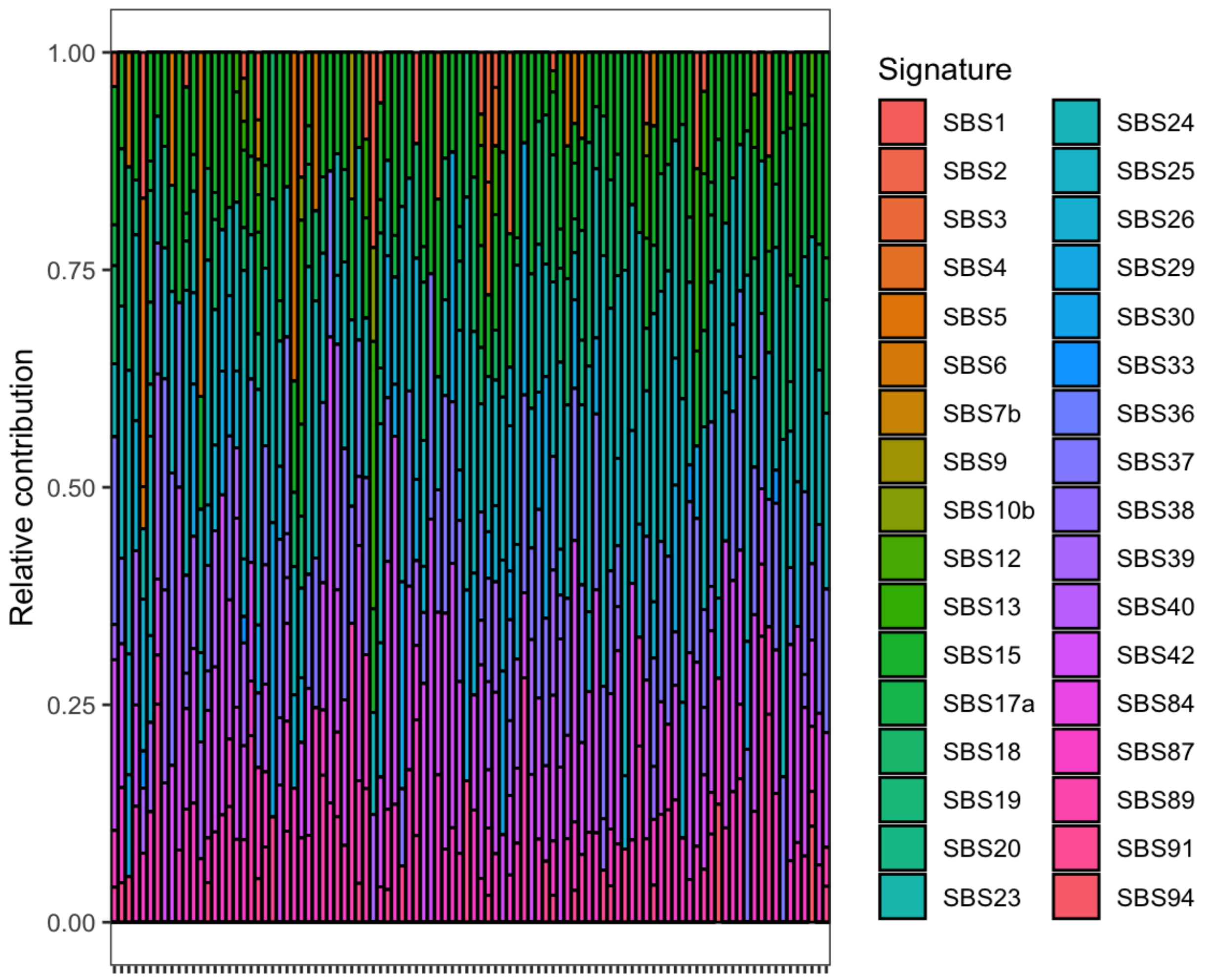

Figure S3. Plot of the relative contribution of fitted COSMIC single-base substitution mutational signatures in each sample.

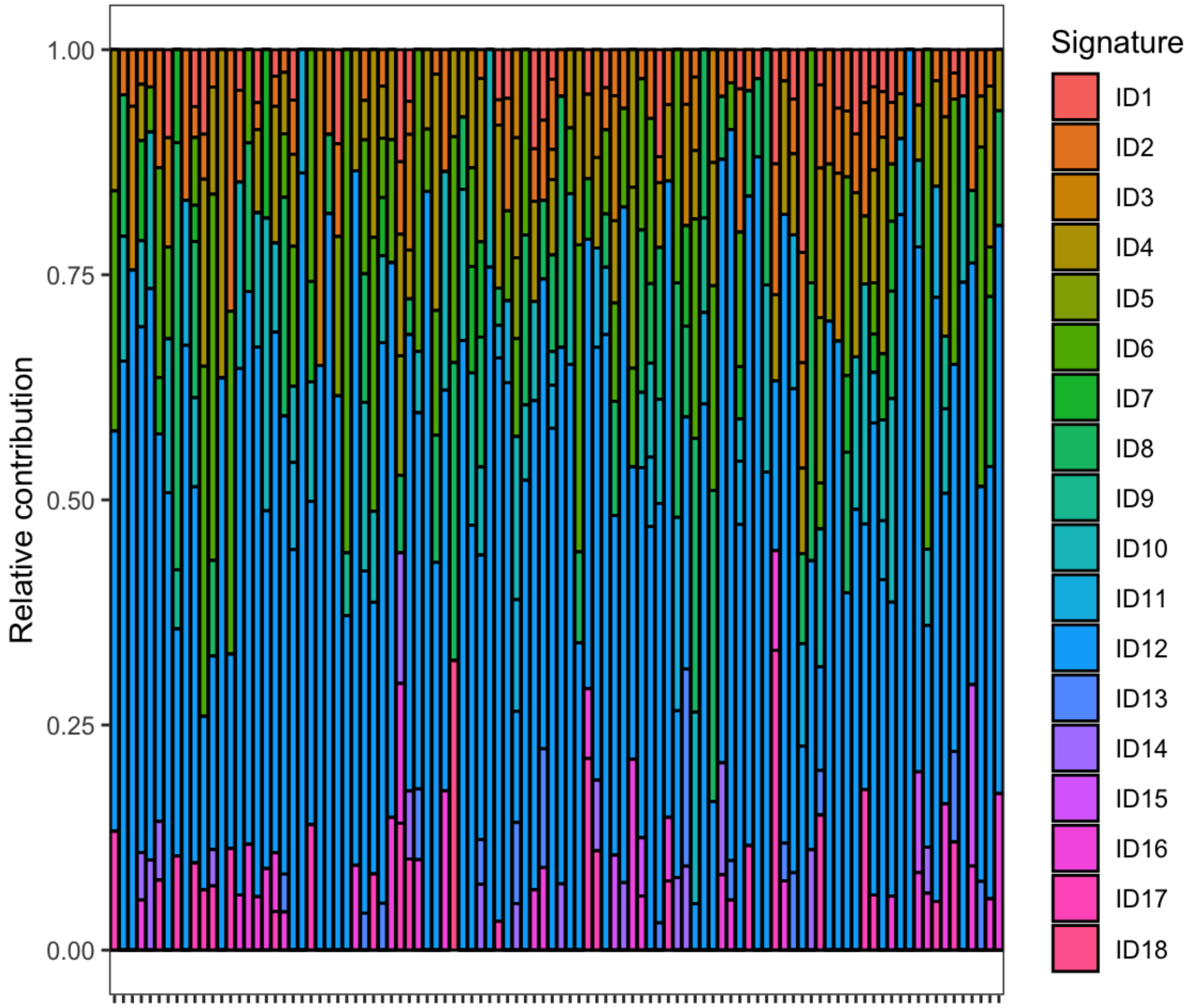

Figure S4. Plot of the relative contribution of fitted COSMIC insertion-deletion mutational signatures in each sample.
